# Single nucleotide polymorphisms of HSP90AA1 gene influence response of SLE patients to glucocorticoids treatment

**DOI:** 10.1186/s40064-016-1911-4

**Published:** 2016-02-29

**Authors:** Yan-Feng Zou, Jian-Hua Xu, Yuan-Yuan Gu, Fa-Ming Pan, Jin-Hui Tao, De-Guang Wang, Sheng-Qian Xu, Hui Xiao, Pei-Ling Chen, Shuang Liu, Jing Cai, Li Lian, Sheng-Xiu Liu, Chun-Mei Liang, Guo Tian, Qian-Ling Ye, Hai-Feng Pan, Hong Su, Dong-Qing Ye

**Affiliations:** Department of Epidemiology and Biostatistics, School of Public Health, Anhui Medical University, 81 Meishan Road, Hefei, 230032 Anhui China; Department of Rheumatology and Immunology, The First Affiliated Hospital of Anhui Medical University, Hefei, 230022 Anhui China; Department of Rheumatology and Immunology, Anhui Medical University Affiliated Provincial Hospital, Hefei, 230001 Anhui China; Department of Nephrology, The Second Affiliated Hospital of Anhui Medical University, Hefei, 230601 Anhui China; Institute of Dermatology and Department of Dermatology, The First Affiliated Hospital of Anhui Medical University, Hefei, 230022 Anhui China; Department of Laboratory Medcine, School of Public Health, Anhui Medical University, Hefei, 230032 Anhui China; Department of Hematology, The Second Affiliated Hospital of Anhui Medical University, Hefei, 230601 Anhui China

**Keywords:** Systemic lupus erythematosus, Glucocorticoids, Heat shock protein 90, Single nucleotide polymorphisms

## Abstract

Heat shock protein 90 (HSP90) is an important glucocorticoid receptor (GR) chaperone protein, and is supposed to be the key factor in regulating glucocorticoids (GCs) effects. The aim of the present study was to explore whether single nucleotide polymorphisms (SNPs) within HSP90AA1 gene affect the response of systemic lupus erythematosus (SLE) patients to GCs treatment. Two hundred and forty-five SLE patients were treated with GCs (prednisone) for 12 weeks. SLE disease activity index (SLEDAI) was used to assess the response of SLE patients to GCs treatment, and patients were classified into sensitive group and insensitive group. HapMap database and Haploview software were used to select tag SNPs. Tag SNPs were genotyped by using multiplex SNaPshot method. Univariate and multivariate logistic regression analyses were used to discriminate the impact of SNPs of HSP90AA1 gene on the response of SLE patients to GCs treatment. Two hundred and thirty three SLE patients finished the 12-week follow-up. Of these patients, 128 patients were included in sensitive group, and 105 patients were included in insensitive group. Seven tag SNPs were selected within HSP90AA1 gene. We detected significant associations for rs7160651 (dominant model: crude OR 0.514, 95 % CI 0.297–0.890, *P* = 0.018; adjusted OR 0.518, 95 % CI 0.293–0.916, *P* = 0.024), rs10873531 (dominant model: crude OR 0.516, 95 % CI 0.305–0.876, *P* = 0.014; adjusted OR 0.522, 95 % CI 0.304–0.898, *P* = 0.019) and rs2298877 (dominant model: crude OR 0.543, 95 % CI 0.317–0.928, *P* = 0.026, adjusted OR 0.558, 95 % CI 0.323–0.967, *P* = 0.037) polymorphisms, but not for other polymorphisms (*P* > 0.05). The present study demonstrates that HSP90AA1 gene SNPs may affect the response of SLE patients to GCs treatment.

## Background

Systemic lupus erythematosus (SLE) is a systemic autoimmune disease characterized by multiorgan dysfunction including neural, cardiovascular, pulmonary, renal, musculoskeletal and cutaneous systems (Rahman and Isenberg 2008; Chalayer et al. 2014). The etiology of SLE is partially known, and multiple genetic and environmental factors are involved (Moser et al. 2009). The ascertainment of SLE prevalence and incidence faces difficulty, and the results of studies on prevalence (range from 20 to 70 per 100,000 persons) and incidence (range from 1 to 10 per 100,000 person-years) of SLE in the general population show marked differences (Borchers et al. 2010; Pons-Estel et al. 2010). The number of patients with SLE exceeds 250,000 in the United States (Rahman and Isenberg 2008). In the past few decades, the life expectancy of SLE patients has improved from an approximate 4-year survival rate of 50 % to a 15-year survival rate of 80 % (Rahman and Isenberg 2008; Merrell and Shulman 1955; Abu-Shakra et al. 1995). However, SLE patients often suffer long-term morbidity that can adversely affect their ability to work as well as quality of life (Pons-Estel et al. 2010). The direct and indirect costs of SLE are substantial among working-age adults, especially in female.

Glucocorticoids (GCs) are currently the mainstay of SLE therapy. Generally, the majority of patients with SLE respond favorably to GCs. However, a lot of evidence of wide interindividual differences in GC efficacy and toxicity among patients have been observed (van Rossum and van den Akker 2011; De Iudicibus et al. 2011). The mechanisms by which interindividual differences develop are not yet fully understood. Genetic factors could, in part, explain the interindividual differences (Cronstein 2006). The effects of GCs are mediated through glucocorticoid receptor (GR), which, a transcription factor, binds steroid hormone ligands to affect the transcription of a number of target genes (Ito et al. 2006; Adcock et al. 1999). The transcriptional activity of GR is regulated by every step of its activation, including ligand binding, transcriptional cofactor binding, nuclear translocation, and DNA binding (Charmandari et al. 2008; Silverman and Sternberg 2008; Ouyang et al. 2012). Heat shock protein 90 (HSP90) is an important GR-related chaperone protein. It is supposed to be the key factor in regulating GCs effects and is essential for activated GR translocation as well as transactivation (Grad and Picard 2007; Murphy 2005).

The HSP90AA1 gene is located on the long arm in human chromosome 14 and a number of single nucleotide polymorphisms (SNPs) of GR gene have been described (Chen et al. 2005). Our previous study has showed that GR genetic variations may play a major role in the response of SLE patients to GCs treatment (Zou et al. 2013b). However, to our knowledge, these is no study to explore the assciation bewteen HSP90AA1 genetic polymorphisms and the response to GCs in patients with SLE. Therefore, in this study, we enrolled 245 SLE patients treated with GCs (prednisone), and assessed the response of SLE patients to GCs treatment. HSP90AA1 gene SNPs were genotyped by using multiplex SNaPshot method. We explored whether SNPs within HSP90AA1 gene are related to the response of Chinese SLE patients to GCs treatment.

## Results and discussion

### Baseline characteristics of patients, genotype frequencies and results of Hardy–Weinberg equilibrium

Two hundred and thirty-three patients (95.10 %) completed the 12-week follow-up. Of these patients, 128 were classified as GCs-sensitive, while 105 patients were classified as GCs-insensitive, including twenty patients used other immunosuppressive agents because of lack of efficacy. No significant difference was found in sex (male: 7.03 vs 6.67 %), age (32.69 ± 11.27 vs 34.16 ± 11.09), body mass index (BMI) (20.76 ± 2.74 vs 21.17 ± 3.50), marital status (married: 72.66 vs 76.19 %), smoking (4.69 vs 1.90 %), alcohol drinking (10.94 vs 14.29 %), baseline SLE disease activity index (SLEDAI) score (11.92 ± 1.91 vs 11.50 ± 2.69) and GCs dose (45.55 ± 14.90 vs 42.57 ± 17.06) between GCs-sensitive group and GCs-insensitive group (Table 1, *P* > 0.05). The genotype frequencies of HSP90AA1 gene 7 tag SNPs were evaluated and were reported in Table 2. The genotype distributions of these SNPs were in Hardy–Weinberg equilibrium (*P* > 0.05). Twelve patients failed to attend scheduled clinic visits. The clinical and demographic characteristics of the dropouts did not differ significantly from the completers (*P* > 0.05).

**Table 1:** Characteristics of of study patients

Characteristic	Sensitive (n = 128)	Insensitive (n = 105)	Overall (n = 233)	*P* value
Sex, No. (%)				0.913
Male	9 (7.03)	7 (6.67)	16 (6.87)	
Female	119 (92.97)	98 (93.33)	217 (93.13)	
Age, mean (SD)	32.69 (11.27)	34.16 (11.09)	33.35 (11.19)	0.318
BMI, mean (SD)	20.76 (2.74)	21.17 (3.50)	20.95 (3.10)	0.333
Marital status, No. (%)				0.539
Unmarried	35 (27.34)	25 (23.81)	60 (27.75)	
Married	93 (72.66)	80 (76.19)	173 (74.25)	
Smoking, No. (%)				0.246
No	122 (95.31)	103 (98.10)	225 (96.57)	
Yes	6 (4.69)	2 (1.90)	8 (3.43)	
Drinking, No. (%)				0.441
No	114 (89.06)	90 (85.71)	204 (87.55)	
Yes	14 (10.94)	15 (14.29)	29 (12.45)	
SLEDAI, mean (SD)	11.92 (1.91)	11.50 (2.69)	11.73 (2.30)	0.174
GCs dose mg/d, mean (SD)	45.55 (14.90)	42.57 (17.06)	44.21 (15.94)	0.157

**Table 2:** Genotype frequencies of single nucleotide polymorphisms in HSP90AA1 gene

Polymorphisms (Minor allele)	Sensitive (n = 128)			Insensitive (n = 105)			Overall (n = 233)			HWE *P* value
	Wild type	Heterozygous	Homozygous mutants	Wild type	Heterozygous	Homozygous mutants	Wild type	Heterozygous	Homozygous mutants	
rs2298878 (A)	121 (94.53)	7 (5.47)	0 (0)	97 (92.38)	8 (7.62)	0 (0)	218 (93.56)	15 (6.44)	0 (0)	0.612
rs7157967 (C)	123 (96.09)	5 (3.91)	0 (0)	103 (98.10)	2 (1.90)	0 (0)	226 (97.00)	7 (3.00)	0 (0)	0.816
rs7160651 (A)	72 (56.25)	50 (39.06)	6 (4.69)	75 (71.43)	26 (24.76)	4 (3.81)	147 (63.09)	76 (32.62)	10 (4.29)	0.964
rs10873531 (G)	61 (47.66)	59 (46.09)	8 (6.25)	67 (63.81)	31 (29.52)	7 (6.67)	128 (54.94)	90 (38.63)	15 (6.43)	0.877
rs1190597 (G)	107 (83.59)	19 (14.85)	2 (1.56)	94 (89.53)	10 (9.52)	1 (0.95)	201 (86.26)	29 (12.45)	3 (1.29)	0.112
rs11547523 (G)	111 (86.72)	16 (12.50)	1 (0.78)	94 (89.52)	11 (10.48)	0 (0)	205 (87.98)	27 (11.59)	1 (0.43)	0.913
rs2298877 (T)	68 (53.13)	53 (41.41)	7 (5.46)	71 (67.62)	27 (25.71)	7 (6.67)	139 (59.66)	80 (34.33)	14 (6.01)	0.585

### Association of HSP90AA1 gene polymorphisms with response of SLE patients to GCs

Univariate logistic analysis showed that the following three HSP90AA1 gene tag SNPs were significantly associated with the response of SLE patients to GCs treatment: rs7160651 (dominant model: OR 0.514, 95 % CI 0.297–0.890, *P* = 0.018), rs10873531 (dominant model: OR 0.516, 95 % CI 0.305–0.876, *P* = 0.014) and rs2298877 (dominant model: OR 0.543, 95 % CI 0.317–0.928, *P* = 0.026). In the multivariate regression analysis, the three SNPs were still significantly associated with the response of SLE patients to GCs treatment (rs7160651 dominant model: OR 0.518, 95 % CI 0.293–0.916, *P* = 0.024; rs10873531 dominant model: OR 0.522, 95 % CI 0.304–0.898, *P* = 0.019; rs2298877 dominant model: OR 0.558, 95 % CI 0.323–0.967, *P* = 0.037). The results of univariate and multivariate logistic regression analyses are shown in Table 3.

**Table 3:** Analysis of glucocorticoids efficacy and single nucleotide polymorphisms of HSP90AA1 gene

Polymorphisms	Dominant model				Recessive model			
	Crude OR (95 % CI)	Crude P value	Adjusted OR (95 % CI)	Adjusted P value	Crude OR (95 % CI)	Crude P value	Adjusted OR (95 % CI)	Adjusted P value
rs2298878	1.426 (0.499–4.070)	0.508	1.508 (0.516–4.405)	0.453	−	−	−	−
rs7157967	0.478 (0.091–2.515)	0.384	0.380 (0.065–2.212)	0.282	−	−	−	−
rs7160651	0.514 (0.297–0.890)	0.018	0.518 (0.293–0.916)	0.024	0.805 (0.221–2.932)	0.743	0.946 (0.251–3.564)	0.935
rs10873531	0.516 (0.305–0.876)	0.014	0.522 (0.304–0.898)	0.019	1.072 (0.375–3.059)	0.897	1.259 (0.425–3.729)	0.678
rs1190597	0.596 (0.273–1.301)	0.194	0.562 (0.251–1.259)	0.162	0.606 (0.054–6.775)	0.684	0.473 (0.040–5.611)	0.553
rs11547523	0.764 (0.341–1.712)	0.513	0.795 (0.349–1.813)	0.586	−	−	−	−
rs2298877	0.543 (0.317–0.928)	0.026	0.558 (0.323–0.967)	0.037	1.235 (0.419–3.639)	0.702	1.429 (0.466–4.380)	0.532

In the present study, we evaluated the effects of SNPs within HSP90AA1 gene on the efficacy of GCs treatment for SLE patients. We observed significant associations of HSP90AA1 gene rs7160651, rs10873531 and rs2298877 polymorphisms with GCs efficacy in Chinese patients with SLE. Our results demonstrate that HSP90AA1 gene polymorphisms may play a role in the response of SLE patients to GCs treatment.

GCs have well-known immunosuppressive effects and are one of the most widely used anti-inflammatory agents in SLE treatment. Despite the proven effectiveness and extensive use, interindividual differences in the response of SLE patients to GCs treatment have been reported, and some patients do not achieve complete remission, or else improve very slowly (van Rossum and van den Akker 2011; De Iudicibus et al. 2011). Meanwhile, the potential for many serious adverse events, including metabolic disease, osteoporosis and increased risk of cardiovascular disease mar the clinical use of GCs (Saag 2002; de Vries et al. 2007; Wang et al. 2012). Patients, especially for those who respond poorly to GCs, are at high risk of adverse events. GCs cause their effects by binding to GR, which is localized in the cytoplasm of target cells, and after binding the ligand, GR-ligand complexes are constructed and translocated into the nucleus (Ito et al. 2006; Adcock et al. 1999). The function of GR is crucially dependent on interactions with HSP90 which facilitates GCs binding to the GR (Grad and Picard 2007; Murphy 2005).

Similarities in the pathologies of cancer and autoimmune diseases, including SLE, have been noted for many years (Eck et al. 2009). HSP90 has been widely studied in cancer. New evidence continues to suggest that HSP90 is overexpressed and associated with poor prognosis in many cancers. Wang et al. (2013) estimated the association of HSP90 expression with clinicopathological parameters, prognosis and the alteration of HSP90 expression after neoadjuvant chemotherapy in patients with advanced gastric cancer. The results showed that HSP90 expression was significantly associated with tumor site, tumor size, depth invasion, lymph node metastasis as well as clinical stages, and the patients with HSP90-positive had worse prognosis than those patients with HSP90-negative. Their study suggested that HSP90 plays an important role on tumor prognosis and may act as a promising target for prognostic prediction in gastric cancer. Tanaka et al. (2013) characterized the efficacy of a potent HSP90 inhibitor NXD30001 against neurofibromatosis type 2 (NF2)-related tumors in vitro and in vivo. Their study showed that HSP90 inhibition has significant antitumor activity against NF2-related tumor cells in vitro and in vivo, and provided certain evidence for the benefit of the HSP90 inhibition against NF2-related tumors. Chu et al. (2013) estimated the role of HSP90AA1 in ovarian cancer and their study suggested that HSP90AA1 is required for the proliferation and survival of SKOV3 cells. They found that HSP90AA1 RNAi could inhibit the proliferation and increase the apoptosis of ovarian cancer SKOV3 cell line. They also found that high expression of HSP90AA1 could partially rescue the survival rate of SKOV3 cells which were treated with cisplatin and decrease the chemosensitivity to cisplatin of SKOV3 cells. Recently, Coskunpinar et al. (2014) investigated HSP90AA1 gene polymorphisms in patients with non-small cell lung cancer (NSCLC). They found that the frequency of mutant genotypes for HSP90AA1 gene rs4947 polymorphism was significantly higher in the patient group than the frequency in controls, indicating that HSP90AA1 gene polymorphisms may contribute to NSCLC development. In SLE patients, high expression of HSP90 has also been detected and correlated with increased levels of IL-6 as well as presence of autoantibodies to HSP90, suggesting that targeting HSP90 may be an effective treatment for SLE (Shukla and Pitha 2012; Hu et al. 2006). In MRL/lpr mouse model of SLE, Shimp et al. (2012) estimated if HSP90 inhibition would reduce disease, and their results suggested that HSP90 may play a role in regulating T cell differentiation as well as activation and that inhibition of HSP90 may reduce inflammation in SLE. Moreover, a recent study has suggested that the accumulation of HSP90 in the nucleus potentially hinders DNA-binding activity and transactivation, which may contribute to GCs resistance in patients with idiopathic nephrotic syndrome (INS) (Ouyang et al. 2012). The above evidence appears to support our resluts. Since HSP90 protein expression may have an impact on cellular sensitivity to systemic GCs treatment, SNPs within HSP90AA1 gene may contribute to decreased/increased GC responsiveness. However, these is no other study to explore the association of HSP90AA1 gene polymorphisms with the response of SLE patients to GCs treatment. Further studies are still needed to assess the current results. The association may result from the direct effect of these polymorphisms themselves, or through linkage disequilibrium with another functional polymorphisms in the structural or regulatory regions of the gene. Further studies of the function of these polymorphisms are needed.

The sample size of the current study is limited. Twelve patients dropped out of the study. Additionally, the use of hydroxychloroquine, considered as a second-line treatment in SLE patients, offers a wide range of benefits (Costedoat-Chalumeau et al. 2010; Chen et al. 2015). Thus, patients also received hydroxychloroquine therapy in the study. These limitations may have certain impact on our results.

## Conclusion

In conclusion, this is the first study to explore the association of the response to GCs with HSP90AA1 genetic polymorphisms in SLE patients. The present study demonstrates that HSP90AA1 gene rs7160651, rs10873531 and rs2298877 polymorphisms may affect the response of SLE patients to GCs treatment. Larger studies are required to further assess the association. Meanwhile, further studies of the function of these polymorphisms are needed.

## Methods

### Subjects and study design

In order to interpert genetic and environmental influence on individualized response to treatment in SLE patients, a pharmacogenetic study was conducted in Anhui Medical University. This study was carried out as part of the pharmacogenetic study. A total of 245 patients, enrolled in the First Affiliated Hospital and the Second Affiliated Hospital of Anhui Medical University (Anhui, China), were included in the study. All patients were of unrelated Han Chinese ancestry, and met the revised criteria for the classification of SLE established by the American College of Rheumatology (ACR) in 1997 (Hochberg 1997). These patients have the SLEDAI score of 5 or higher, and no patients had recent exposure to GCs (in the last three months). Potential patients were excluded if they: (1) had lupus crisis or required GCs plus therapy; (2) had systemic fungal or bacterial infection; (3) were pregnant or lactating women; (4) were allergic to hydroxychloroquine. The treatment and efficacy assessment used for the present study has been described in our previous study (Zou et al. 2013a, b). Briefly, patients were treated with GCs (prednisone) combined with hydroxychloroquine (400 mg/day) for 12 weeks. At the start of the study, prednisone 10 mg/day–0.5 mg/kg/day was administrated to the patients whose SLEDAI scores were <10, and 0.5–1 mg/kg/day was administrated to the patients whose SLEDAI scores were ≥10. The dosage adjustments were made through the consultation from the rheumatologists during the course of treatment. SLEDAI was used to assess the response of SLE patients to GCs treatment at weeks 0, 4, 8 and 12. The GCs-sensitive was defined as a 4 points or less in the SLEDAI score at the 12 weeks. The GCs-insensitive was defined as a 5 points or greater in the SLEDAI score at the 12 weeks, and patients who took other immunosuppressive agents because of lack of efficacy in the course of treatment were also considered as GCs-insensitive. The ethical committee of Anhui Medical University approved the study. We obtained written informed consent from all patients and fully explained the procedures of the study.

### Tag SNP selection and genotyping

HapMap database and Haploview software were used to select tag SNPs. SNPs within HSP90AA1 gene were retrieved from HapMap database (Chinese Han in Beijing) sample (release No. 24/phaseII Nov08, on NCBI B36 assembly). r^2^ threshold of 0.8 and minor allele frequency (MAF) threshold of 0.01 were used as cut-off for tag SNPs selection, and, finally, 7 tag SNPs were selected: rs2298878, rs7157967, rs7160651, rs10873531, rs1190597, rs11547523 and rs2298877. Venous blood was obtained by venepuncture from each subject. We extracted DNA from the peripheral blood by using a blood extraction kit (QIAGEN, Germany), and stored DNA at –80 °C until use. Genotyping of tag SNPs in HSP90AA1 gene was performed by multiplex SNaPshot technology using an ABI fluorescence-based assay allelic discrimination method (Applied Biosystems, Foster city, CA).

### Statistical analysis

Mean and standard deviation (SD) of continuous variables, and proportions of categorical variables are presented as descriptive statistics. Differences for continuous variables were tested by using *t*-test. Chi square test was used to test differences for categorical variables and Hardy–Weinberg equilibrium. Dominant and recessive models were applied for the analysis of genotype distribution. Univariate and multivariate logistic regression analyses were used to discriminate the impact of SNPs of HSP90AA1 gene on the response of SLE patients to GCs treatment (GCs-sensitive 0, GCs-insensitive 1). We used multivariate logistic regression to adjust for potential confounding factors (sex, age, BMI, marital status, smoking status, alcohol drinking, baseline SLEDAI score and GCs dose (mg/day)). All statistical analyses were performed using *SPSS* software version 10.01 (SPSS, Inc, Chicago, IL). Within this report, *P* < 0.05 were considered statistically significant.

## Abbreviations

SLE: systemic lupus erythematosus
GCs: glucocorticoids
GR: glucocorticoid receptor
HSP90: heat shock protein 90
SNPs: single nucleotide polymorphisms
BMI: body mass index
SLEDAI: SLE disease activity index
NF2: neurofibromatosis type 2
NSCLC: non-small cell lung cancer
INS: idiopathic nephrotic syndrome
ACR: American College of Rheumatology
MAF: minor allele frequency
SD: standard deviation
